# A Real World 10-Year Experience With Vascular Closure Devices and Large-Bore Access in Patients Undergoing Transfemoral Transcatheter Aortic Valve Implantation

**DOI:** 10.3389/fcvm.2021.791693

**Published:** 2022-01-21

**Authors:** Gregor Heitzinger, Christina Brunner, Sophia Koschatko, Varius Dannenberg, Katharina Mascherbauer, Kseniya Halavina, Carolina Doná, Matthias Koschutnik, Georg Spinka, Christian Nitsche, Markus Mach, Martin Andreas, Florian Wolf, Christian Loewe, Christoph Neumayer, Michael Gschwandtner, Andrea Willfort-Ehringer, Max-Paul Winter, Irene M. Lang, Philipp E. Bartko, Christian Hengstenberg, Georg Goliasch

**Affiliations:** ^1^Division for Cardiology, Department of Internal Medicine II, Medical University of Vienna, Vienna, Austria; ^2^Department of Cardiothoracic Surgery, Medical University of Vienna, Vienna, Austria; ^3^Department of Cardiovascular and Interventional Radiology, Medical University of Vienna, Vienna, Austria; ^4^Department of Vascular Surgery, Medical University of Vienna, Vienna, Austria; ^5^Division for Angiology, Department of Internal Medicine II, Medical University of Vienna, Vienna, Austria

**Keywords:** TAVR, vascular closure device, vascular complication, VARC III, bleeding, hematoma, pseudoaneurysm, sheath size

## Abstract

Transcatheter aortic valve replacement (TAVR) has established itself as a safe and efficient treatment option in patients with severe aortic valve stenosis, regardless of the underlying surgical risk. Widespread adoption of transfemoral procedures led to more patients than ever being eligible for TAVR. This increase in procedural volumes has also stimulated the use of vascular closure devices (VCDs) for improved access site management. In a single-center examination, we investigated 871 patients that underwent transfemoral TAVR from 2010 to 2020 and assessed vascular complications according to the Valve Academic Research Consortium (VARC) III recommendations. Patients were grouped by the VCD and both, vascular closure success and need for intervention were analyzed. In case of a vascular complication, the type of intervention was investigated for all VCDs. The Proglide VCD was the most frequently used device (*n* = 670), followed by the Prostar device (*n* = 112). Patients were old (median age 83 years) and patients suffered from high comorbidity burden (60% coronary artery disease, 30% type II diabetes, 40% atrial fibrillation). The overall rate of major complications amounted to 4.6%, it was highest in the Prostar group (9.6%) and lowest in the Manta VCD group (1.1% *p* = 0.019). The most frequent vascular complications were bleeding and hematoma (*n* = 110, 13%). In case a complication occurred, 72% of patients did not need any further intervention other than manual compression or pressure bandages. The rate of surgical intervention after complication was highest in the Prostar group (*n* = 15, 29%, *p* = 0.001). Temporal trends in VCD usage highlight the rapid adoption of the Proglide system after introduction at our institution. In recent years VCD alternatives, utilizing other closure techniques, such as the Manta device emerged and increased vascular access site management options. This 10-year single-center experience demonstrates high success rates for all VCDs. Despite successful closure, a significant number of patients does experience minor vascular complications, in particular bleeding and hematoma. However, most complications do not require surgical or endovascular intervention. Temporal trends display a marked increase in TAVR procedures and highlight the need for more refined vascular access management strategies.

## Introduction

Since its first description in 2002 (1), transcatheter aortic valve replacement (TAVR) has been established as a safe and efficient treatment for patients with severe aortic valve stenosis and high surgical risk (2). Recent studies emphasize the efficacy of TAVR in patients with low and intermediate surgical risk (3, 4).

This has led to drastic increase in procedural volumes over the last years, with TAVR now exceeding the numbers of surgical aortic valve replacement (SAVR) (5). For 2019, the TS-ACC TVT Registry (Society of Thoracic Surgeons–American College of Cardiology Transcatheter Valve Therapy Registry) reports 72 991 TAVR procedures in the US alone. Similar trends can also be observed in Europe (6). Additionally, the recently published guidelines for valvular heart disease extended the recommendation for TAVR to patients ≥75 years or those who are at high surgical risk (STS-PROM/ EuroSCORE II > 8%) for the treatment of severe aortic stenosis (Class I recommendation) (7). With societal affirmation, more patients than ever are now eligible for TAVR.

Transfemoral TAVR procedures require insertion of large arterial sheaths for TAVR system guidance and delivery. Ongoing development and device refinement led to significant decrease of delivery system size, resulting in higher procedural safety and success. Nonetheless, vascular and access—related complications remain a setback in the TAVR success. In order to standardize endpoint definition in TAVR, the first Valve Academic Research Consortium (VARC) guidelines were published in 2011, of which a third revised version has recently been published with updated and newly proposed endpoints and definitions (8).

While percutaneous vascular closure devices (VCD) have been used regularly for peripheral vascular, percutaneous coronary and rhythmological interventions (9), application of VCDs to large bore arteriotomies as in the setting of TAVR is more challenging. However, percutaneous closure was shown to be associated with a decrease in access- site infections, lower bleeding complications and shorter hospital stays in comparison to surgical cut-down techniques (10). Several VCDs utilizing various closure techniques have been developed to improve access site management and to decrease vascular complications (11, 12).

The aim for the present analysis was to investigate the utilization of VCDs in every day clinical practice, assess temporal trends in VCD usage and to provide real-world experience of VCDs and vascular complications.

## Methods

We included patients with severe aortic stenosis that were referred to TAVR or valve-in-valve procedures by our local Heart Team at the Medical University of Vienna, a tertiary care center. The evaluation of vascular complications was performed retrospectively including baseline procedural, clinical characteristics, procedural protocols, discharge letters and femoral ultrasound reports. All patients received a femoral ultrasound after transfemoral access for TAVR according to standard operating procedures. To assess temporal trends, we choose our study period to be from 2010 to 2020. This study was approved by the institutional review board of the Medical University of Vienna.

### TAVR Procedure

All patients underwent computed tomography before implantation and were evaluated for femoral access. Choice of femoral access site was based on arterial diameter and qualitative interpretation of vessel tortuosity and calcium and plaque burden by the operator. Patients in which transfemoral access was not feasible were excluded from this study. After arterial puncture, stepwise dilatation of the access site was performed until the delivery system was inserted. The standard secondary access site at our institution is the contralateral femoral vein (5Fr sheath) and the contralateral femoral artery with a 6Fr sheath. Vascular closure devices were deployed according to the manufacturer's recommendations and successful closure was confirmed by angiogram and assessment of hemostasis by the operator. Anticoagulation during the procedure was achieved using weight adapted unfractionated heparin and guided by the activated clotting time. Every patient received a postprocedural femoral ultrasound before discharge.

### Vascular and Access-Site-Related Complications According to VARC III Criteria

Every complication was classified according to VARC III recommendations, additionally the number of complications for each patient was recorded. Successful vascular closure was defined as achievement of hemostasis, using vascular closure devices and manual compression and or planned adjunctive endovascular balloon dilatation after retraction of TAVR delivery systems. Hemostasis was assessed by the operator. Patients that experienced vascular complications were evaluated whether any intervention was necessary or possible according to the recommendations of angiologists, interventional radiologists and vascular surgeons. The type of intervention was categorized into surgical, endovascular (stents or angioplasty) or other type of interventions, encompassing thrombin instillation, ultrasound-guided compression and coilembolization and no intervention at all, other than regular follow up in an outpatient setting, manual compression or pressure bandages.

Briefly, the VARC-III criteria for vascular and access-site-related complications include complications that are in direct relation to the vascular access site, manipulation of delivery devices (including puncture needles, wires and catheters) or the delivery process, but not implantation of the valve itself. However, also complications at any accessory vascular access (contralateral arterial/venous access, radial access) site should be reported as vascular complications. Also, any complications that may occur in the postprocedural phase need to be considered (site infection, pseudoaneurysm). Another potential source of complication that has gained recognition over the last few years are closure device failures, defined as the inability to achieve hemostasis at the vascular access site, with the need to revert to other forms of treatment. Of note, manual compression and endovascular balloon dilatation are not included in this definition.

Generally, major and minor complications should be differentiated. Major complications lead to death, amputation, limb or visceral ischemia, irreversible neurologic or end-organ damage or ≥ VARC 2 bleeding, while minor vascular complications do not result in the above-mentioned endpoints.

### Vascular Closure Devices

Several vascular closure devices are currently available utilizing various forms of closure techniques, ranging from suture-based to collagen or membrane-based systems (9, 13). The device selection criteria are based on availability, vessel diameter, calcification and operator familiarity and preference.

The Prostar XL (Abbott cardiovascular, Santa Clara, CA, USA) is a suture-based vascular closure device designed for complete percutaneous vascular closure of large bore arteriotomies that uses active approximation. This 10-French device is advanced over the guidewire until the needles are in the arterial lumen. If positioned correctly, a pulsatile blood flow will exit the device. Then four nitinol needles forming two suture loops are deployed, which are then secured with a sliding knot and a knot pusher (14).

The PerClose Proglide (Abbott cardiovascular, Santa Clara, CA, USA) is a suture-based vascular closure device. Depending on the access site one or two devices can be used and it utilizes a preclose technique. The systems are advanced over a guidewire and sutures are deployed before insertion of the TAVR delivery system. The preclose technique involves two Proglide devices, that are rotated 30° from the midline of the arterial access and deployed at 10 o‘clock and 2 o‘clock (15). After removal of the arterial sheath, the sutures are tightened until hemostasis is achieved. If necessary, further sutures can be deployed over the guidewire and fastened with a knot pusher (13).

In contrast to the above-mentioned suture-based systems, the Manta (Essential Medical Inc., Exton, PA, USA) system is a collagen-based VCD designed for arteriotomies created from devices ranging from 12-French to 25-French. The device features a hemostatic collagen plug on the outside of the arterial wall, that is anchored by a small polymer toggle on the inner side of the vessel wall. These components are secured in a sandwich-type manner by a small metallic lock (11).

### Statistics

Categorical data are presented as count and percentage and compared using the Chi-square or Fisher's exact test as appropriate. For numerical data median and interquartile range (IQR) were used to display distribution and Kruskal-Wallis test for comparison. Additionally, we performed univariate logistic regression analysis to assess clinical predictors for vascular complications within the VCD groups. Four patients received other VCDs than the Prostar, Proglide or Manta. These were excluded from the overall cohort. The R software [R Core Team (2020). R: A language and environment for statistical computing. R Foundation for Statistical Computing, Vienna, Austria. URL https://www.R-project.org/.] was used for all analyses and data visualization.

## Results

### Baseline Characteristics

Baseline clinical characteristics are displayed in Table 1, stratified according to the VCD used. In total, data on closure devices and vascular complications was available in 871 patients, 47% (409) of which were male. Proglide system was used in 670 patients, followed by Prostar VCD in 112 and Manta in 89 patients. Patients were generally older (median age 83) and suffered from high comorbidity burden, reflected by a median EuroSCORE II of 4.1%. Almost 60% of patients had a history of coronary artery disease and type II diabetes was present in nearly 30% in all VCD groups. Overall VCD groups showed good homogeneity, although Prostar patients were significantly older than the other VCD groups (*p* = 0.003). Median hemoglobin values were also lower in Prostar patients (10.9 g/dL, *p* = 0.002) compared to the rest. Regarding the antithrombotic discharge medication, 46% received single antiplatelet therapy, 13% were on any P2Y12 agent and 30% were prescribed oral anticoagulation. Additionally, 95 patients were discharged with a triple antithrombotic regimen. There were no significant differences among the VCD subgroups. Table 2 depicts a summary of TAVR prosthesis and French size of delivery systems. The Edwards Sapien S3 was the most frequently used valve (*n* = 343, 39%), followed by the Boston Scientific Acurate Neo (predecessor models by Symetis were included) (*n* = 141, 16%). Patients that received Edwards Sapien/ Sapien XT valve models were also mainly treated with the Prostar VCD (*n* = 100, 89%). In patients with larger delivery systems, the Prostar device was used most frequently (74%), while smaller sheath sizes mainly used the Proglide System. Nearly half of the procedures (*n* = 413, 47%) were fluoroscopic guided roadmap punctures, of which 122 patients had one or more vascular complications in contrast to 174 patients in the non-fluoroscopic guided roadmap punctures group (*n* = 458, *p* = 0.01).

**Table 1:** Baseline clinical characteristics according to used closure device.

**Characteristic**	**Overall, *N* = 871^a^**	**Proglide, *N* = 670^a^**	**Manta, *N* = 89^a^**	**Prostar, *N* = 112^a^**	***P*-value^b^**
Sex, male	409 (47%)	307 (46%)	48 (54%)	54 (48%)	0.3
Body mass index, kg/m2	26.1 (23.4–29.4)	26.1 (23.4–29.4)	27.4 (23.8–30.1)	25.1 (22.6–28.4)	**0.042**
Age, years	83 (78–86)	82 (78–86)	81 (78–86)	84 (81–87)	**0.003**
EuroSCORE II	4.1 (3.9–4.5)	4.1 (3.9–4.5)	4.1 (3.9–4.4)	4.1 (3.8–4.4)	0.7
STS score	4.2 (3.8–4.8)	4.1 (3.8–4.6)	4.3 (4.0–5.1)	NA	0.14
Coronary artery disease	516 (59%)	391 (58%)	53 (60%)	72 (64%)	0.5
Atrial fibrillation	358 (41%)	275 (41%)	35 (39%)	48 (43%)	0.9
Hypertension	770 (88%)	587 (88%)	81 (91%)	102 (91%)	0.4
Peripheral vascular disease	91 (10%)	68 (10%)	10 (11%)	13 (12%)	0.9
Cerebral vascular disease	111 (13%)	90 (13%)	9 (10%)	12 (11%)	0.5
COPD^*^	109 (13%)	89 (13%)	7 (7.9%)	13 (12%)	0.3
Diabetes, type II	269 (31%)	207 (31%)	27 (30%)	35 (31%)	>0.9
Hypercholesterinemia	520 (60%)	398 (60%)	64 (72%)	58 (52%)	**0.015**
Current smoker	52 (6.8%)	43 (7.5%)	5 (6.0%)	4 (3.8%)	0.4
Hemoglobin, g/dL	11.7 (10.2–12.9)	11.7 (10.3–12.9)	11.9 (10.8–13.1)	10.9 (10.1–12.2)	**0.002**
Hematokrit, %	34.9 (31.3–38.3)	35.0 (31.4–38.6)	35.5 (32.1–38.6)	33.3 (30.1–36.5)	**0.004**
Discharge antithrombotic agents					0.3
Aspirin, single therapy	395 (46%)	309 (46%)	35 (39%)	51 (46%)	
Oral anticoagulant	263 (30%)	205 (31%)	23 (26%)	35 (31%)	
P2Y12 agent	115 (13%)	83 (12%)	19 (21%)	13 (12%)	
Triple antithrombotic regimen	95 (11%)	70 (10%)	12 (13%)	13 (12%)	

a *n (%); Median (IQR)*,

b *Pearson's Chi-squared test; Kruskal-Wallis rank sum test*.

* *Chronic obstructive pulmonary disease*.

*Bold values indicate statistical significance*.

**Table 2:** Baseline prosthesis characteristics according to vascular closure device used.

**Characteristic**	**Overall, *N* = 871^a^**	**Proglide, *N* = 670^a^**	**Manta, *N* = 89^a^**	**Prostar, *N* = 112^a^**
**Valve used**				
Abbott Portico	103 (12%)	103 (15%)	0 (0%)	0 (0%)
Boston Scientific Accurate Neo	141 (16%)	92 (14%)	49 (55%)	0 (0%)
Edwards Sapien S3	343 (39%)	303 (45%)	36 (40%)	4 (3.6%)
Edwards Sapien XT	122 (14%)	22 (3.3%)	0 (0%)	100 (89%)
Medtronic Corevalve	12 (1.4%)	5 (0.7%)	0 (0%)	7 (6.2%)
Medtronic Corevalve Evolut R	98 (11%)	97 (14%)	1 (1.1%)	0 (0%)
Medtronic Corevalve Evolut R Pro	22 (2.5%)	22 (3.3%)	0 (0%)	0 (0%)
Other	30 (3.4%)	26 (3.9%)	3 (3.4%)	1 (0.9%)
**Valve delivery system size, fr**				
14	420 (49%)	353 (53%)	64 (72%)	3 (2.7%)
16	252 (29%)	153 (23%)	17 (19%)	82 (74%)
>16	188 (22%)	154 (23%)	8 (9.0%)	26 (23%)

a *n (%)*.

### Vascular Access Site Complications and Vascular Closure Device

Overall number of major complications was 4.6% (*n* = 39) and most frequent in the Prostar group with a total of 10 patients (9.6%, *p* = 0.019). Over a timespan of 10 years, 259 minor complications occurred as displayed in Table 3. In comparison to the other VCD groups, minor vascular complications were more frequent in the Prostar group (*n* = 43, 38%, *p* = 0.051). The most frequent complications were bleeding and hematoma (*n* = 110, 13%), closely followed by dissection and pseudoaneurysm (*n* = 106, 12%). 219 patients (25%) had one vascular complication, and 77 (8.8%) had ≥ two vascular complications, results were similar for each VCD. Successful hemostasis after TAVR was achieved in nearly all patients, closure rates were however significantly lower in the Prostar group 93% (*n* = 99, *p* = 0.016).

**Table 3:** Vascular complications according to the main vascular closure device used.

**Characteristic**	**Overall, *N* = 871^a^**	**Proglide, *N* = 670^a^**	**Manta, *N* = 89^a^**	**Prostar, *N* = 112^a^**	***P*-value^b^**
**VARC III major vascular complication**	39 (4.6%)	28 (4.2%)	1 (1.1%)	10 (9.6%)	**0.019**
**VARC III minor vascular complication**	259 (30%)	186 (28%)	30 (34%)	43 (38%)	0.051
**Type of vascular complication**					
Arterial or venous thrombosis/occlusion/stenosis/ischemia	52 (6.0%)	42 (6.3%)	7 (7.9%)	3 (2.7%)	
Bleeding or hematoma	110 (13%)	65 (9.7%)	10 (11%)	35 (31%)	
Closure device failure or unplanned intervention	3 (0.3%)	1 (0.1%)	0 (0%)	2 (1.8%)	
Dissection or pseudoaneurysm	106 (12%)	86 (13%)	9 (10%)	11 (9.8%)	
Perforation, rupture or fistula	25 (2.9%)	19 (2.8%)	5 (5.6%)	1 (0.9%)	
**Number of complications**					**0.031**
No vascular complication	575 (66%)	457 (68%)	58 (65%)	60 (54%)	
One vascular complication	219 (25%)	157 (23%)	25 (28%)	37 (33%)	
≥2 vascular complication	77 (8.8%)	56 (8.4%)	6 (6.7%)	15 (13%)	
**Successful achievement of hemostasis after procedure**	837 (97%)	650 (98%)	88 (100%)	99 (93%)	**0.016**
**Need for intervention**	83 (40%)	58 (39%)	9 (47%)	16 (38%)	0.8
**Type of intervention**					**0.001**
Surgical intervention	48 (16%)	32 (15%)	1 (3.2%)	15 (29%)	
Endovascular intervention	29 (9.8%)	22 (10%)	7 (23%)	0 (0%)	
Other	6 (2.0%)	4 (1.9%)	1 (3.2%)	1 (1.9%)	
**Number of periprocedural RBC transfusions, 400 ml**					0.2
0	836 (96%)	643 (96%)	88 (99%)	105 (94%)	
1	9 (1.0%)	9 (1.3%)	0 (0%)	0 (0%)	
≥2	26 (3.0%)	18 (2.7%)	1 (1.1%)	7 (6.2%)	

a *n (%)*,

b *Fisher's exact test; Pearson's Chi-squared test*.

*Bold values indicate statistical significance*.

### Vascular Access Site Complications and Management According to Closure Devices

**Figure 1:**
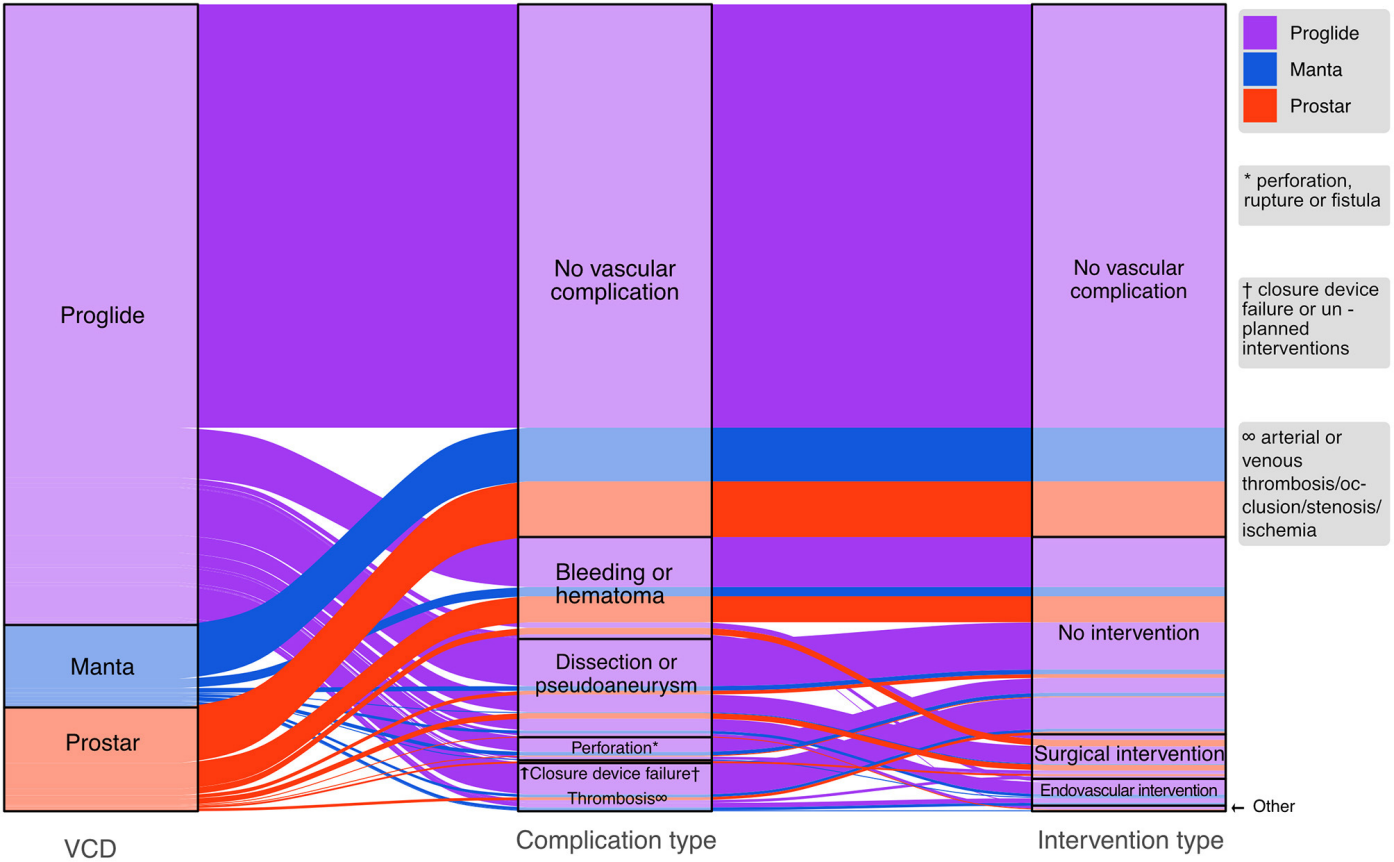
Alluvial diagram displaying the proportion of utilized vascular closure devices (VCD) (left part) and the vascular complications that occurred within each VCD group (middle part). The right part indicates how each vascular complication was treated.

If a complication occurred, there were significant differences in the management depending on VCD subgroup. In the Manta group, endovascular interventions were more common (*n* = 7, 23%, *p* = 0.001), while complications in the Prostar device group were more likely to need surgical revision (*n* = 15, 29%, *p* = 0.001). Of the 29 patients that were treated with endovascular interventions, 15 underwent balloon angioplasty and 14 were stented. A combined strategy, utilizing VCD and Angioseal (St. Jude Medical, St. Paul, MN, USA), was adopted in 69 patients, of which 29% experienced a vascular access complication. Regarding the location of vascular complications, the majority (67%) of patients had the complication at the primary vascular access point, while 23% experienced the complication at a secondary access point and 10% had vascular complication at the primary and secondary access site. Figure 1 depicts an alluvial diagram, that visualizes the frequency and distribution of VCDs, the complication type and whether an intervention was needed for treatment.

The results of univariable logistic regression analysis are displayed in Table 4. Briefly, in the Proglide subgroup male patients were less likely to have any vascular complication [OR 0.58, CI: (0.41–0.81], *p* = 0.001) and in the Manta group an increase in BMI was associated with a reduced risk for vascular complications [OR 0.90, CI: (0.81–0.99), *p* = 0.039].

**Table 4:** Results of univariable logistic regression assessing the risk factors for any vascular complication (major and minor combined) in each VCD subgroup separately.

**Predictor**	**Proglide**		**Manta**		**Prostar**	
	**Odds ratio (95% CI)**	***P*-value**	**Odds ratio (95% CI)**	***P*-value**	**Odds ratio (95% CI)**	***P*-value**
Sex, male	0.58 (0.41–0.81)	**0.001**	1.11 (0.45–2.74)	0.81	0.49 (0.22–1.07)	0.079
Body mass index, kg/m2	0.99 (0.95–1.02)	0.576	0.90 (0.81–0.99)	**0.039**	0.95 (0.86–1.03)	0.235
Age, years	1.00 (0.98–1.03)	0.432	0.97 (0.91–1.04)	0.433	1.06 (0.98–1.16)	0.119
EuroSCORE II	0.96 (0.80–1.04)	0.574	0.62 (0.21–1.04)	0.284	1.00 (0.69–1.46)	0.978
STS score	0.79 (0.50–1.12)	0.243	0.88 (0.42–1.05)	0.595	NA	
Coronary artery disease	1.01 (0.72–1.41)	0.946	0.73 (0.29–1.81)	0.498	1.95 (0.87–4.47)	0.105
Atrial fibrillation	0.83 (0.59–1.16)	0.283	0.95 (0.38–2.37)	0.924	1.73 (0.79–3.84)	0.167
Hypertension	1.51 (0.90–2.63)	0.127	0.33 (0.06–1.62)	0.172	3.89 (0.88–27.01)	0.101
Peripheral vascular disease	1.50 (0.88–2.52)	0.13	0.47 (0.07–2.05)	0.363	0.83 (0.25–2.71)	0.767
Cerebral vascular disease	0.76 (0.45–1.23)	0.274	2.86 (0.70–12.48)	0.14	2.18 (0.64–8.64)	0.228
COPD^*^	1.27 (0.79–2.02)	0.319	0.31 (0.02–1.97)	0.296	0.34 (0.07–1.24)	0.124
Diabetes, type II	0.85 (0.59–1.22)	0.399	0.35 (0.11–0.99)	0.061	1.09 (0.47–2.56)	0.830
Hypercholesterinemia	0.91 (0.65–1.27)	0.583	0.97 (0.36–2.74)	0.963	1.47 (0.68–3.21)	0.327
Current smoker	1.04 (0.52–1.98)	0.91	9.17 (1.27–184.44)	0.053	3.26 (0.40–67.35)	0.313

* *Chronic obstructive pulmonary disease*.

*Bold values indicate statistical significance*.

**Figure 2:**
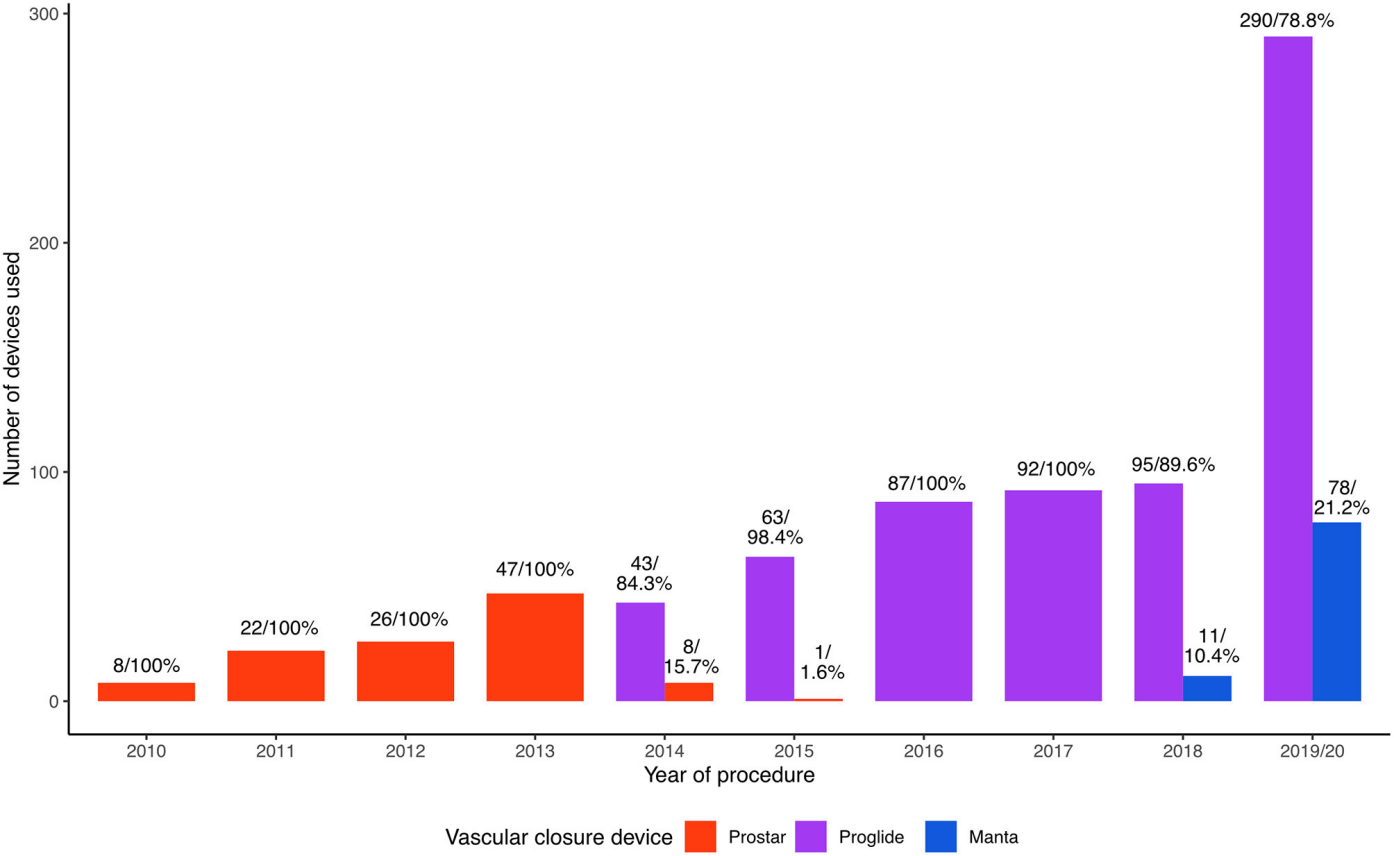
Temporal trends of vascular closure device (VCD) utilization. In the first few years the only used device was the Prostar device. Additionally, rapid adoption of the Proglide (purple) system after its introduction can be observed.

Figure 2 depicts the number of VCD used per study year, highlighting the increase in procedural volumes of TAVR in the last decade. The first few years saw high utilization of the Prostar VCD, however, after its introduction, the Proglide system rapidly became the tool of choice.

## Discussion

In this study, we present a single-center real-world 10-year experience with vascular closure devices and vascular access complications in patients undergoing transfemoral TAVR. Our main finding is that the vascular closure devices used in this study (Proglide, Prostar, Manta) are generally safe and offer successful closure after TAVR procedure in the vast majority of patients. However, despite large procedural volumes, vascular complications, in particular bleeding and hematoma, still affect a considerable number of patients. Nevertheless, most complications do not need surgical or endovascular intervention. More options in vascular closure devices help to diversify and to expand the range of treatment to allow a personalized approach to vascular closure.

### Temporal Trends and Evolution of Closure Devices

The results of this study delineate two major changes in temporal trends. Firstly, the main target population of TAVR did significantly change within the last 10 years. From initially high surgical risk to nowadays low-to intermediate-risk patients, a wide variety of patients can now be treated with TAVR. Additionally, an increase in VCD options has provided the operator with more flexibility, than in the early 2010s.

The Prostar device was the main vascular closure device used in the first few years of the study period and was associated with higher complication rate in comparison to later developed devices in regard to minor and major vascular complications. Results of prior studies comparing the Prostar to the Proglide system are inconsistent and conflicting. A retrospective study of 278 patients conducted in 2015 reports significantly lower rate of vascular complications and closure device failure for the Prostar group (16), while the authors of a study with 585 patients undergoing transfemoral TAVR, conclude the use of Proglide resulted in significantly lower rate of major and minor bleedings and VCD failures (17). This is supported by data from a recent prospective multicenter study, (18) and a randomized trial where the authors investigated the impact of VCDs on bleeding in patients receiving either Unfractionated Heparin or Bivalirudin (9). This trend is also reflected in our experience, additionally we observed significantly higher rates of surgical intervention after vascular complications with the Prostar device (Figure 1). However, it is important to note that patients treated with Prostar were higher surgical risk patients and TAVR technology, in particular sheath sizes, were not as refined and small, likely influencing the number of vascular complications. Proposed mechanisms for this difference in performance are the Prostar's larger size (10Fr vs. 6Fr) and larger separation of subcutaneous tissue around the arterial wall. Also, differences in the suture deployment are likely to play a role (17).

We found no clear benefit of using the Manta device over the Proglide system, however as visible in Figure 2, the Manta was only utilized in most recent years. Moriyama and colleagues conducted a propensity score matched analysis in 2019 in 111 patients. They report a significantly lower bleeding complication rate for the Manta device, while overall rate of vascular complications was similar between the two VCD groups (19). In this study cohort, VARC III major vascular complications were significantly lower in the Manta group (*n* = 1, 1.1% vs. *n* = 28, 4.2%) than in patients, where Proglide was used. Rate of minor vascular complications was slightly higher in Manta patients (*n* = 28, 32% vs. *n* = 184, 28%, *p* = 0.051), and the type of complications showed a similar distribution. This is in contrast to data from the recently published CHOICE-CLOSURE trial (Randomized Comparison of CatHeter-based Strategies fOr Interventional ACcess SitE CLOSURE during Transfemoral Transcatheter Aortic Valve Implantation), comparing the Manta plug-based system to the suture-based Proglide, with the option for a small plug. 258 patients were randomized to the Manta system and demonstrated a shorter time to hemostasis, but significantly higher rate of vascular access complications and bleeding (20).

### Future Aspects for Vascular Access Closure After TAVR

Future studies will need to investigate potential clinical, laboratory or morphologic predictors of successful vascular closure after large—bore arteriotomies. Other studies found higher age, implantation and VCD failure to be predictors of major VARC III complications (12). In contrast, our data indicate no association to increased vascular complications in higher aged patients for all VCD groups. This finding might reflect that rather the biological vessel age including plaque burden, calcium load, fibrotic remodeling and tortuosity might be more important than patient age is in risk stratification. Previous literature also found female gender to be independent predictors of vascular complications (21, 22). This is in part supported by the present findings as males in the Proglide subgroups were less likely have vascular complications (23). Identification of predictors for unsuccessful closure and vascular complications will help to refine access site management strategies. Additionally, CT-derived morphologic parameters have shown to be predictive of vascular complications and are likely to enhance risk-stratification for vascular access related complications (22, 24–26). Further research will need to investigate patients at high risk for vascular complications and other methods of access site management, such as planned surgical closure.

This 10-year experience provides a unique real-world insight into the utilization of VCDs. Over this period of time, the overall increase in procedural volumes can be observed. Additionally, refinement of technology and device improvements led to rapid decline in superseded products. Most recently, the introduction of a collagen-based closure device for large bore access closure adds an additional tool to the armamentarium of closure devices utilizing a complementary technique. This development now offers health-care professionals more options for vascular closure to optimize access site management approach. With the increasing number of TAVR procedures in recent years and more patients than ever being eligible for transfemoral access, an increased variety in VCD option is needed to ensure a personalized approach to large bore access closure.

## Limitations

This study has several strengths and limitations. The aim of this study was to report a comprehensive review on temporal trends of VCD utilization over a substantial period of time. As VCDs populations were not matched, conclusions regarding the comparison of VCD performances are not possible by design. Furthermore, the TAVR patient population changed within the study period as a consequence of guideline recommendations. In addition, preoperative CT (computed tomography) scan derived quantifiable parameters are likely to further augment risk-stratification and prediction of vascular complications However, the inclusion of CT parameters was beyond the scope of this study. Furthermore, there is certain learning curve to every new VCD. Although, this may be a confounding factor, we only have a small number of TAVR operators at our institution, therefore limiting the number of learning phases and the duration of the study period extends far beyond any learning phase, likely mitigating the influence of learning curves on the overall trend. This study represents a real-world experience with VCDs and vascular complication management of a large all-comer study cohort and provides long term insights.

## Conclusion

This single-center experience with various vascular closure devices over a time span of 10 years, indicates that success rates are high for all VCDs, yet a significant number of patients experience minor vascular complications, in particular bleeding and hematoma. However, complications mostly do not require surgical or endovascular intervention. Temporal trends display a drastic increase in TAVR procedures and highlight the need for more refined vascular access management strategies.

## Data Availability Statement

The raw data supporting the conclusions of this article will be made available by the authors, without undue reservation.

## Author Contributions

GH, M-PW, PB, GG, and CH: contributed to the study design. CB, SK, VD, KM, and KH: contributed to data analysis and data collection. GG, GH, and PB: conducted the statistical analysis. GH: wrote the first draft. CD, MK, GS, CNi, MM, GG, and PB: contributed to manuscript revision. MA, FW, CL, CNe, MG, AW-E, IL, CH, and GG: did oversee the study planning and organization and performed the procedures. All authors contributed to the article and approved the submitted version.

## Funding

The authors declare that this study received funding from Edwards, Abbott, Medtronic, LSI. The funder was not involved in the study design, collection, analysis, interpretation of data, the writing of this article or the decision to submit it for publication.

## Conflict of Interest

MA has received institutional research funding (Edwards, Abbott, Medtronic, LSI) and has served as a proctor/speaker/consultant (Edwards, Abbott, Medtronic). The remaining authors declare that the research was conducted in the absence of any commercial or financial relationships that could be construed as a potential conflict of interest.

## Publisher's Note

All claims expressed in this article are solely those of the authors and do not necessarily represent those of their affiliated organizations, or those of the publisher, the editors and the reviewers. Any product that may be evaluated in this article, or claim that may be made by its manufacturer, is not guaranteed or endorsed by the publisher.

## Abbreviations

CT: computed tomography
SAVR: surgical aortic valve replacement
TAVR: transcatheter aortic valve replacement
TS-ACC TVT Registry: Society of Thoracic Surgeons–American College of Cardiology Transcatheter Valve Therapy Registry
VARC III: Valve Academic Research Consortium III
VCD: vascular closure device.

## References

[1] Cribier A Eltchaninoff H Bash A Borenstein N Tron C Bauer F. Percutaneous transcatheter implantation of an aortic valve prosthesis for calcific aortic stenosis: first human case description. Circulation. (2002) 106:3006–8. 10.1161/01.CIR.0000047200.36165.B8 12473543

[2] Mack MJ Leon MB Smith CR Miller DC Moses JW Tuzcu EM. 5-year outcomes of transcatheter aortic valve replacement or surgical aortic valve replacement for high surgical risk patients with aortic stenosis (PARTNER 1): a randomised controlled trial. Lancet. (2015) 385:2477–84. 10.1016/S0140-6736(15)60308-7 25788234

[3] Leon MB Smith CR Mack MJ Makkar RR Svensson LG Kodali SK. Transcatheter or surgical aortic-valve replacement in intermediate-risk patients. N Engl J Med. (2016) 374:1609–20. 10.1056/NEJMoa1514616 27040324

[4] Mack MJ Leon MB Thourani VH Makkar R Kodali SK Russo M. Transcatheter aortic-valve replacement with a balloon-expandable valve in low-risk patients. N Engl J Med. (2019) 380:1695–705. 10.1056/NEJMoa1814052 30883058

[5] Carroll JD Mack MJ Vemulapalli S Herrmann HC Gleason TG Hanzel G. STS-ACC TVT registry of transcatheter aortic valve replacement. Ann Thorac Surg. (2021) 111:701–22. 10.1016/j.athoracsur.2020.09.002 33213826

[6] Hamm CW Beyersdorf F. GARY-the largest registry of aortic stenosis treatment worldwide. Eur Heart J. (2020) 41:733–5. 10.1093/eurheartj/ehaa048 32031223

[7] Vahanian A Beyersdorf F Praz F Milojevic M Baldus S Bauersachs J. 2021 ESC/EACTS guidelines for the management of valvular heart disease. Eur Heart J. (2021) ehab395. [Epub ahead of print]. 10.1093/eurheartj/ehab395 34931612 PMC9725093

[8] Varc-3 Writing C Genereux P Piazza N Alu MC Nazif T Hahn RT. Valve academic research consortium 3: updated endpoint definitions for aortic valve clinical research. Eur Heart J. (2021) 42:1825–57. 10.1093/eurheartj/ehaa799 33871579

[9] Noori VJ Eldrup-Jorgensen J. A systematic review of vascular closure devices for femoral artery puncture sites. J Vasc Surg. (2018) 68:887–99. 10.1016/j.jvs.2018.05.019 30146036

[10] Nakamura M Chakravarty T Jilaihawi H Doctor N Dohad S Fontana G. Complete percutaneous approach for arterial access in transfemoral transcatheter aortic valve replacement: a comparison with surgical cut-down and closure. Catheter Cardiovasc Interv. (2014) 84:293–300. 10.1002/ccd.25130 23873857

[11] Wood DA Krajcer Z Sathananthan J Strickman N Metzger C Fearon W. Pivotal clinical study to evaluate the safety and effectiveness of the manta percutaneous vascular closure device. Circ Cardiovasc Interv. (2019) 12:e007258. 10.1161/CIRCINTERVENTIONS.119.007258 31296082

[12] Mehilli J Jochheim D Abdel-Wahab M Rizas KD Theiss H Spenkuch N. One-year outcomes with two suture-mediated closure devices to achieve access-site haemostasis following transfemoral transcatheter aortic valve implantation. EuroIntervention. (2016) 12:1298–304. 10.4244/EIJV12I10A213 27866140

[13] Toggweiler S Leipsic J Binder RK Freeman M Barbanti M Heijmen RH. Management of vascular access in transcatheter aortic valve replacement: part 1: basic anatomy, imaging, sheaths, wires, and access routes. JACC Cardiovasc Interv. (2013) 6:643–53. 10.1016/j.jcin.2013.04.003 23866177

[14] Haas PC Krajcer Z Diethrich EB. Closure of large percutaneous access sites using the prostar XL percutaneous vascular surgery device. J Endovasc Surg. (1999) 6:168–70. 10.1177/152660289900600209 10473335

[15] Griese DP Reents W Diegeler A Kerber S Babin-Ebell J. Simple, effective and safe vascular access site closure with the double-ProGlide preclose technique in 162 patients receiving transfemoral transcatheter aortic valve implantation. Catheter Cardiovasc Interv. (2013) 82:E734–41. 10.1002/ccd.25053 23765732

[16] Barbanti M Capranzano P Ohno Y Gulino S Sgroi C Imme S. Comparison of suture-based vascular closure devices in transfemoral transcatheter aortic valve implantation. EuroIntervention. (2015) 11:690–7. 10.4244/EIJV11I6A137 26499222

[17] Seeger J Gonska B Rodewald C Rottbauer W Wohrle J. Impact of suture mediated femoral access site closure with the prostar XL compared to the proglide system on outcome in transfemoral aortic valve implantation. Int J Cardiol. (2016) 223:564–7. 10.1016/j.ijcard.2016.08.193 27561160

[18] Berti S Bedogni F Giordano A Petronio AS Iadanza A Bartorelli AL. Efficacy and safety of proglide versus prostar XL vascular closure devices in transcatheter aortic valve replacement: the RISPEVA registry. J Am Heart Assoc. (2020) 9:e018042. 10.1161/JAHA.120.018042 33103545 PMC7763424

[19] Moriyama N Lindstrom L Laine M. Propensity-matched comparison of vascular closure devices after transcatheter aortic valve replacement using MANTA versus proglide. EuroIntervention. (2019) 14:e1558–e65. 10.4244/EIJ-D-18-00769 30295293

[20] Abdel-Wahab M Hartung P Dumpies O Obradovic D Wilde J Majunke N. Comparison of a pure plug-based versus a primary suture-based vascular closure device strategy for transfemoral transcatheter aortic valve replacement: the CHOICE-CLOSURE randomized clinical trial. Circulation. (2021). [Epub ahead of print]. 10.1161/CIRCULATIONAHA.121.057856 34738828

[21] Van Mieghem NM Tchetche D Chieffo A Dumonteil N Messika-Zeitoun D van der Boon RM. Incidence, predictors, and implications of access site complications with transfemoral transcatheter aortic valve implantation. Am J Cardiol. (2012) 110:1361–7. 10.1016/j.amjcard.2012.06.042 22819428

[22] Dencker D Taudorf M Luk NH Nielsen MB Kofoed KF Schroeder TV. Frequency and effect of access-related vascular injury and subsequent vascular intervention after transcatheter aortic valve replacement. Am J Cardiol. (2016) 118:1244–50. 10.1016/j.amjcard.2016.07.045 27638098

[23] Naddaf A Williams S Hasanadka R Hood DB Hodgson KJ. Predictors of groin access pseudoaneurysm complication: a 10-year institutional experience. Vasc Endovascular Surg. (2020) 54:42–6. 10.1177/1538574419879568 31578127

[24] Ruge H Burri M Erlebach M Lange R. Access site related vascular complications with third generation transcatheter heart valve systems. Catheter Cardiovasc Interv. (2021) 97:325–32. 10.1002/ccd.29095 32588968

[25] Hayashida K Lefevre T Chevalier B Hovasse T Romano M Garot P. Transfemoral aortic valve implantation new criteria to predict vascular complications. JACC Cardiovasc Interv. (2011) 4:851–8. 10.1016/j.jcin.2011.03.019 21851897

[26] Batchelor W Patel K Hurt J Totten J Burroughs P Smith G. Incidence, prognosis and predictors of major vascular complications and percutaneous closure device failure following contemporary percutaneous transfemoral transcatheter aortic valve replacement. Cardiovasc Revasc Med. (2020) 21:1065–73. 10.1016/j.carrev.2020.01.007 31974033 PMC8140523

